# Postnatal development of the small intestinal mucosa drives age-dependent, regio-selective susceptibility to *Escherichia coli* K1 infection

**DOI:** 10.1038/s41598-017-00123-w

**Published:** 2017-03-06

**Authors:** George M. H. Birchenough, Fatma Dalgakiran, Luci A. Witcomb, Malin E. V. Johansson, Alex J. McCarthy, Gunnar C. Hansson, Peter W. Taylor

**Affiliations:** 1 Department of Medical Biochemistry, University of Gothenburg, SE-405 30 Gothenburg, Sweden; 2 School of Pharmacy, University College London, London, WC1N 1AX UK

## Abstract

The strong age dependency of neonatal systemic infection with *Escherichia coli* K1 can be replicated in the neonatal rat. Gastrointestinal (GI) colonization of two-day-old (P2) rats leads to invasion of the blood within 48 h of initiation of colonization; pups become progressively less susceptible to infection over the P2-P9 period. We show that, in animals colonized at P2 but not at P9, *E. coli* K1 bacteria gain access to the enterocyte surface in the mid-region of the small intestine and translocate through the epithelial cell monolayer by an intracellular pathway to the submucosa. In this region of the GI tract, the protective mucus barrier is poorly developed but matures to full thickness over P2-P9, coincident with the development of resistance to invasion. At P9, *E. coli* K1 bacteria are physically separated from villi by the mucus layer and their numbers controlled by mucus-embedded antimicrobial peptides, preventing invasion of host tissues.

## Introduction

*Escherichia coli* is a leading cause of early- and late-onset neonatal sepsis; a high proportion of strains responsible for these life-threatening infections express the K1 capsule, an anti-phagocytic, polysialic acid homopolymer that facilitates survival in the bloodstream and is closely associated with the capacity to invade and cause disease in the central nervous system of susceptible infants^1, 2^. Neonatal *E. coli* K1 infection is critically dependent on vertical transmission of the causative agent from mother to infant^3^. In a minority of colonized neonates, *E. coli* K1 translocates from the gut lumen to the blood and elicits symptoms of sepsis and septic shock^2^. Subsequent invasion of the central nervous system is likely to result in meningeal inflammation which has a major impact on the severity of outcome, even after sites of infection have been sterilized by antibiotic chemotherapy^4^.

There are only limited opportunities to investigate the etiology and pathogenesis of human infections. Fortunately key features, in particular the strong age dependency, can be replicated in the neonatal rat by oral administration of *E. coli* K1, initiating stable colonization of the GI tract in both adult and neonatal animals^5, 6^. For the first few days of life, newborn *E. coli* K1-colonized rat pups are prone to develop lethal systemic infection as colonizing bacteria translocate from the lumen of the GI tract to the blood^6, 7^ from where they may establish infection in multiple organs, including the brain^8, 9^. Invasion of brain tissue induces a strong local inflammatory reaction concomitant with up-regulation of genes associated with the innate immune response^10^. Within a week, the pups become refractory to systemic infection, even in the presence of persistent GI tract colonization^7^.

The basis of the strong age dependency of *E. coli* K1 systemic infection is determined by the capacity of the bacteria to translocate from the GI lumen to the bloodstream *via* the mesenteric lymph nodes^6, 7^. The GI tract is protected along its entire length by mucus with variable properties that prevents the large majority of commensal and pathogenic bacteria from reaching the apical surface of the epithelial layer^11, 12^. The major component of mucus in the small intestine and colon is the large, gel-forming glycoprotein Muc2 secreted by goblet cells^13^. In the small intestine, mucus forms a single porous layer and contains an array of antimicrobial peptides secreted by crypt Paneth and epithelial cells that further enhance its defensive capacity. Colonic mucus is thicker and consists of an inner layer impenetrable to bacteria and an outer layer in which commensal bacteria reside^14^.

Although genes encoding mucus glycoproteins are expressed in embryonic and foetal intestine^15^, GI tissues undergo significant *postpartum* development. In the neonatal rat, substantial changes in developmental gene expression occur between two (P2) and nine (P9) days *postpartum* ^16^, including upregulation of genes encoding antimicrobial α-defensin peptides: following *E. coli* K1 GI colonization, α-defensin genes *defa24* and *defa-rs1* were found to be up-regulated in P9, but not P2, pups. Conversely, developmental expression of Tff2 was massively dysregulated in P2 but not P9 rats and was accompanied by a decrease in the amount of colonic Muc2^16^. These changes will compromise innate GI defenses in susceptible P2 animals but enhance protection in older resistant pups and contribute to the age-related susceptibilities to *E. coli* K1 infection.

In this study, we investigated the impact of postnatal mucus development on systemic infection in the neonatal rat following *E. coli* K1 GI tract colonization. Colonizing bacteria formed an intimate association with the epithelium in the mid-section of the P2 small intestine; they translocated by a transcellular route to the blood *via* the mesenteric lymph nodes in P2, but not P9, animals. There was a strong correlation between susceptibility to systemic infection, the thickness of the mucus layer, and the capacity of bacteria to associate with the epithelial surface. *In vivo* ablation of Paneth cells or *ex vivo* disruption of the mucus layer allowed *E. coli* K1 to invade normally resistant P9 small intestinal tissue, demonstrating cooperation between mucus and antimicrobial secretions in the prevention of *E. coli* K1 invasive infection.

## Results

### Dynamics of colonization and infection of neonatal rats by *E. coli* A192PP

**Figure 1:**
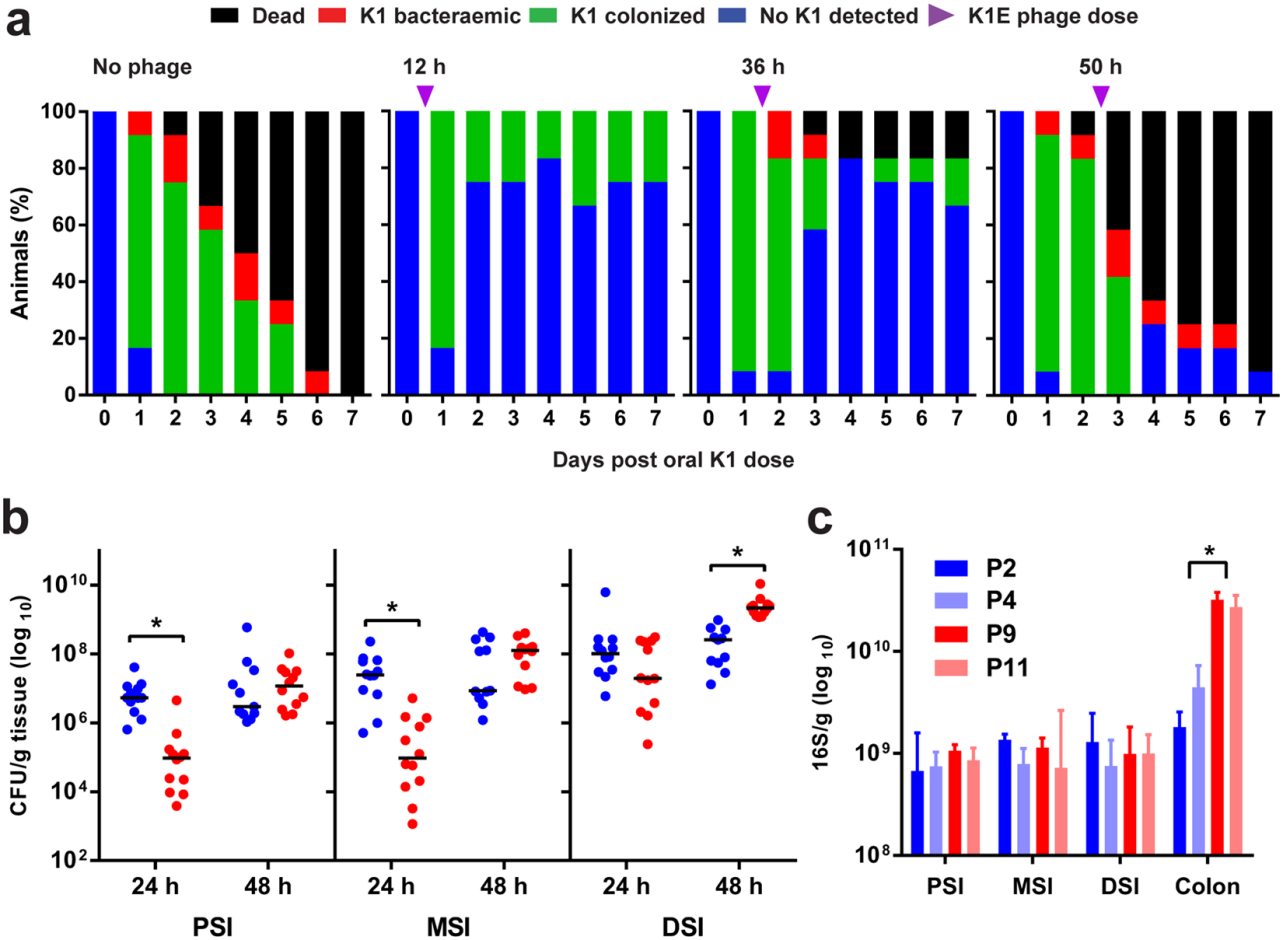
Dynamics of colonization and infection of neonatal rats following an oral dose of *E. coli* K1. (**a**) Survival, colonization and bacteraemic status of neonatal rats dosed orally with 4–8 × 10^6^ CFU *E. coli* A192PP at P2. Colonized animals were fed vehicle buffer (no phage) or 10^9^ PFU K1E bacteriophage 12, 36 or 50 h after receiving colonizing bacteria. *n* = 12 for each group; data from at least two independent experiments. For bacterial quantification, 2 cm segments of proximal (PSI), mid- (MSI) and distal (DSI) small intestine and colon were excised from P2 and P9 neonatal rat pups. Colonization of small intestinal regions 24 and 48 h after oral dosing of neonatal rats with *E. coli* A192PP at P2 or P9 was determined by culture-dependent enumeration of *E. coli* K1 bacteria (**b**); *n* = 4–12 animals; line is median, *P < 0.05. (**c**) Total bacterial load in small intestinal and colonic tissue segments as determined by qPCR of 16S rRNA genes; median ± range, *P < 0.05 (Tukey’s multiple comparison).

Oral administration of *E. coli* K1 strain A192PP to P2 rat pups resulted in GI tract colonization within 24–48 h (Fig. 1a); all animals subsequently developed *E. coli* K1 bacteraemia and succumbed to infection within one week. The incidence of bacteraemia and mortality were significantly reduced in P2 rats receiving an oral dose of *E. coli* K1-specific phage K1E (10^9^ PFU) at 12 or 36 but not 50 h after initiation of colonization (Fig. 1a), indicating that invasion of the blood circulation occurs within the first two days of colonization. We previously determined that oral administration of ~10^6^ CFU *E. coli* A192PP to P2, P5 and P9 pups resulted in a stable GI tract *E. coli* K1 population of 10^7^–10^8^ bacteria/g GI tissue^16^. There were no significant differences between the age groups at any sampling point over a 120 h period, but there were relatively more *E. coli* K1 in the colon of P9 compared to P2 pups.

To examine the spatial distribution in more detail, segments (2 cm) from the proximal (PSI), mid- (MSI) and distal small intestine (DSI) as well as sections of the colon were excised as described earlier^17^. The *E. coli* K1 content of segments from colonized pups was established 24 and 48 h after colonization by viable counting (Fig. 1b) and confirmed by qPCR (*E. coli* K1-specific *neuS* gene; Fig. S1). Significantly more *E. coli* K1 were found in the PSI and MSI from P2 compared to P9 animals 24 h after oral administration of A192PP, although these differences were not evident 24 h later (Fig. 1b and S1). Quantification of the total bacterial load by 16S qPCR in non-colonized neonates demonstrated that the P9 colon contained significantly more bacteria than corresponding tissues from P2 pups. Otherwise, there were no significant differences in the distribution of bacteria along the GI tract (Fig. 1c), indicating that differences in K1 content between P2 and P9 intestinal sections (Fig. 1b and S1) were not due to age-dependent differences in overall bacterial load.

### Impact of colonization by *E. coli* A192PP on GI tract integrity

**Figure 2:**
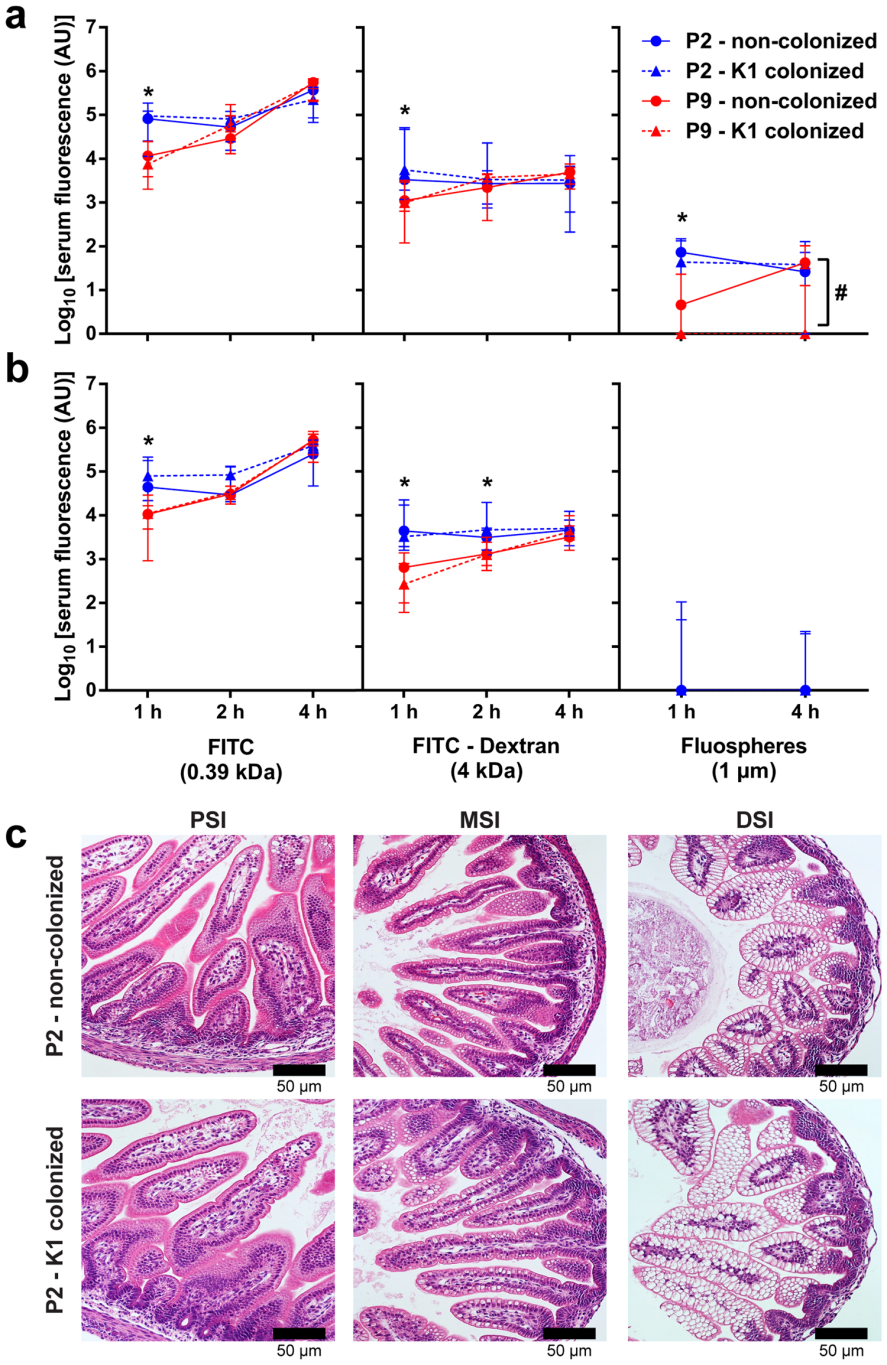
Colonization with *E. coli* K1 does not alter the integrity of the intestinal epithelium. Age dependency of intestinal permeability in non-colonized P2 and P9 pups 24 h (**a**) and 48 h (**b**) after *E. coli* A192PP colonization or sham colonization (broth vehicle alone); plasma fluorescence was determined at indicated time points after oral administration. In addition, neonatal rats were colonized at P2 or P9, fluorescent probes administered orally 24 h or 48 h later and plasma fluorescence determined. Plasma fluorescence was expressed as log_10_ relative fluorescence units (RFU). (**c**) Micrographs of H&E stained PSI, MSI and DSI tissue obtained from neonates 48 h after sham (non-colonized) or A192PP (K1-colonized) feeding at P2. In all experiments, *n* = 6–8 animals; median ± range, n.s., not significant, *P < 0.05, **P < 0.01 (Student’s *t*). ^#^Indicates significant difference between sham-colonized and *E. coli* A192PP-colonized pups.

The mammalian neonatal gut is more permeable to ions, small molecules and macromolecules than the adult gut, a phenomenon that is largely attributable to macropinocytosis of luminal content by neonatal enterocytes^18, 19^. Pathogens may utilise macropinocytosis as a mechanism to invade host cells; thus, age-dependent alterations in intestinal permeability may play a role in the age-dependency of *E. coli* K1 infection. In order to assess differences in permeability over the 48 h infection window we fed non-colonized neonates small molecule (FITC, 389 Da), polymeric (FITC-dextran, 4 kDa), and particulate (1 µm diameter beads) fluorescent probes 24 h (Fig. 2a) and 48 h (Fig. 2b) after P2 and P9. Quantification of serum fluorescence 1, 2 and 4 h after oral administration of the probes showed that probe uptake was more rapid in the P2 neonates, but that the P9 gut was also permeable to the probes. This indicated that the development of resistance to *E. coli* K1 infection over the P2-P9 period was unlikely to be due to developmental alterations in intestinal integrity.

Comparison of serum fluorescence between non-colonized and *E. coli* K1-colonized neonates provided no evidence that colonization induced any increase in intestinal permeability (Fig. 2a,b), indicating that *E. coli* K1 colonization did not cause epithelial cell damage. Permeability to the fluorescent microspheres was at least two to three orders of magnitude lower than for the soluble probes. There was a small but significant decrease in GI permeability 24 h after *E. coli* K1 colonization (Fig. 2a) that was not evident at 48 h, at which point any uptake of the particulate probe was below the level of detection. Lack of damage to the epithelium was supported by examination of H&E-stained PSI, MSI and DSI tissue sections, which revealed a normal neonatal intestinal architecture, including highly vacuolar DSI enterocytes, with no evidence of tissue damage or inflammation (Fig. 2c). Thus, pathogen-induced damage to the epithelial barrier did not play a role in systemic *E. coli* K1 infection.

### Intracellular *E. coli* K1 are found exclusively in mid-small intestine

**Figure 3:**
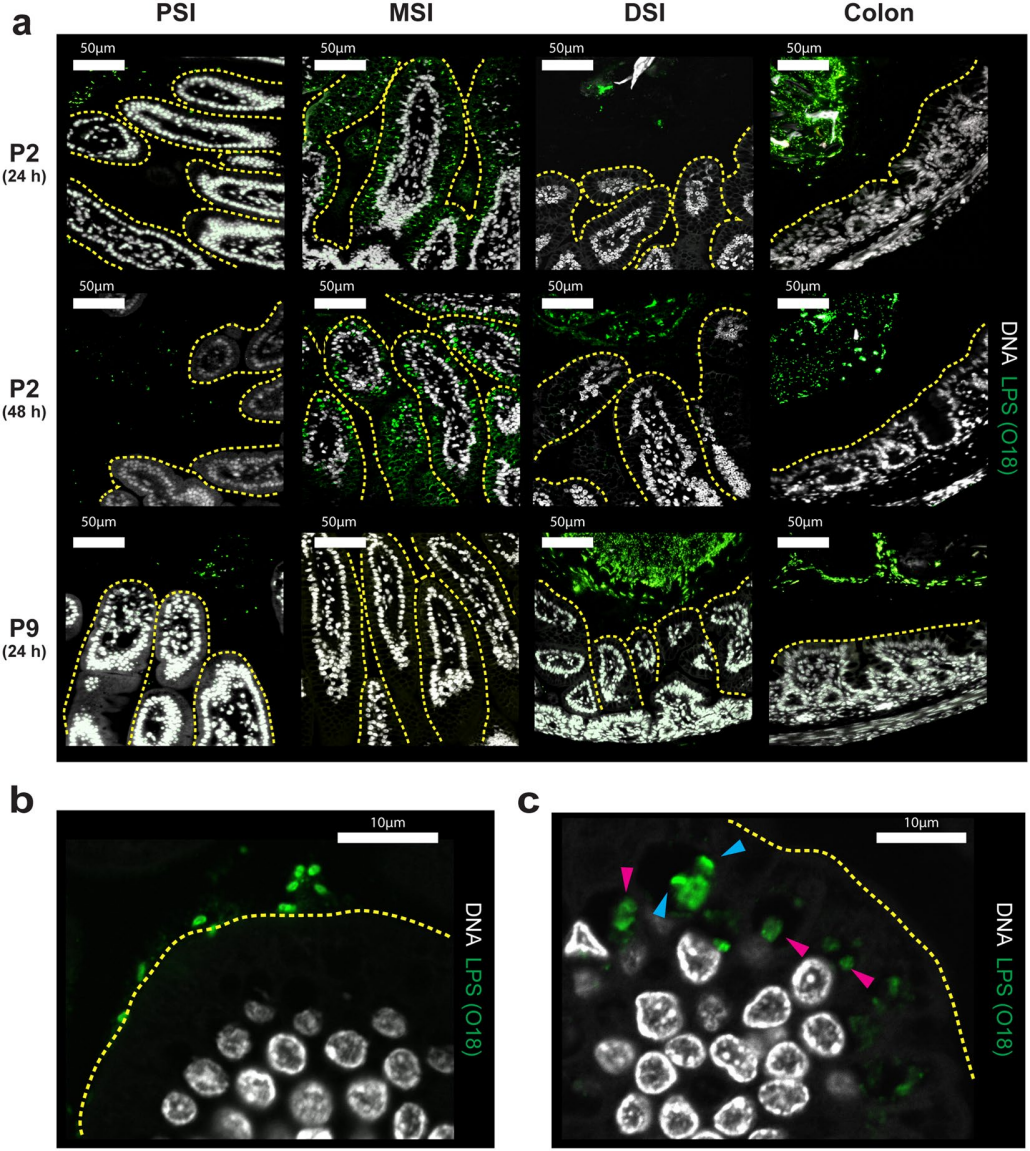
*E. coli* K1 bacteria colonize and invade the MSI of susceptible P2 rat pups. (**a**) Confocal micrographs of Methacarn-fixed PSI, MSI, DSI and colonic tissue sections from neonates 24 and 48 h (as indicated) after oral dosing of *E. coli* A192PP at P2 or P9; sections stained for O18 LPS (green) and DNA (grey); epithelial surface indicated by yellow dashed line. Images are representative of the various regions of the GI tract of at least three colonized pups. (**b**,**c**) Confocal micrographs of MSI tissue from neonatal rats 24 h after oral dosing with *E. coli* K1 at P2; intracellular LPS staining with bacterial cellular morphology () or a more diffuse appearance () indicated.

**Figure 4:**
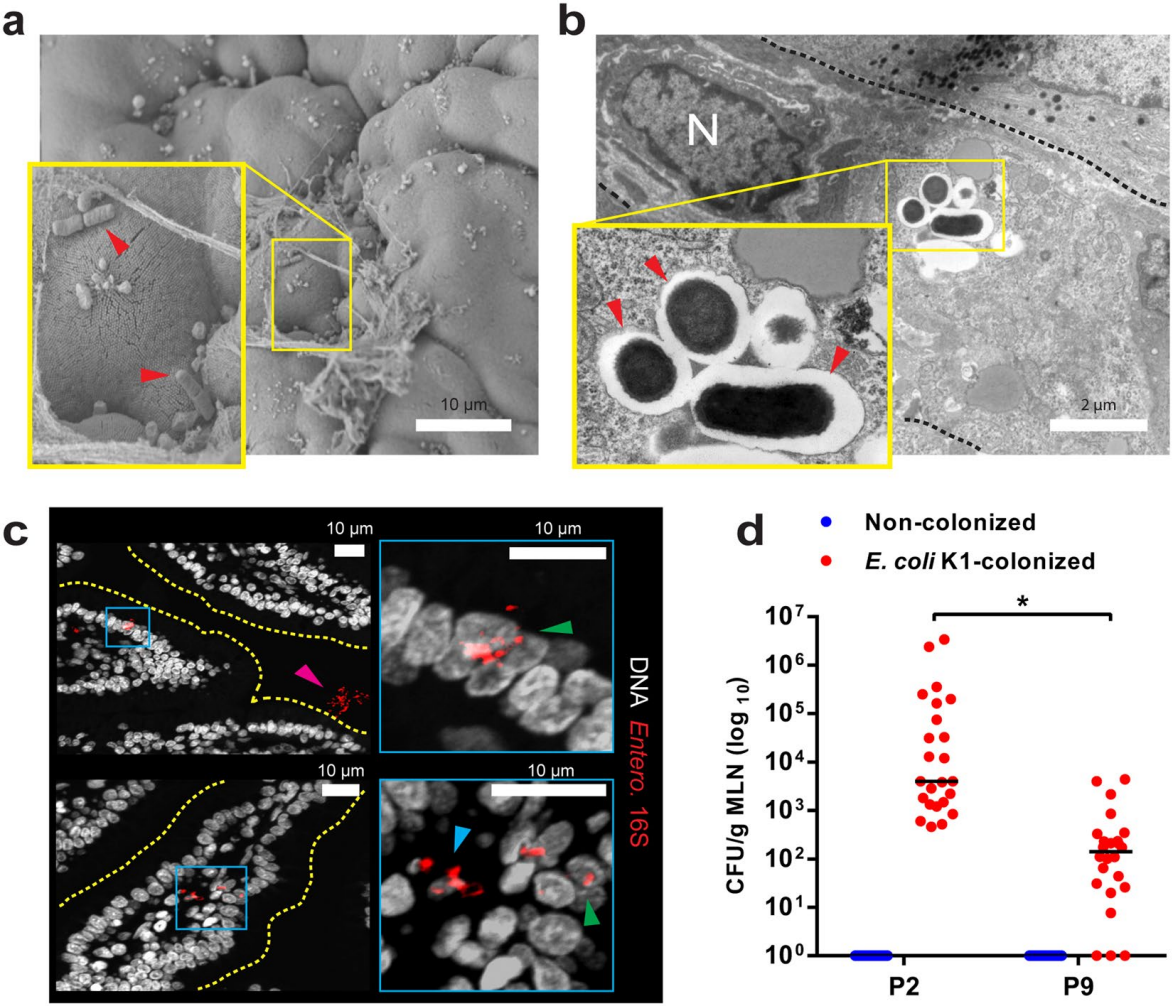
*E. coli* K1 invasion of the MSI epithelium results in translocation of live bacteria to the intestinal mesenteric lymph nodes (**a**) SEM of MSI tissue from neonates 24 h after *E. coli* K1 colonization at P2; tissue-associated bacteria indicated (). (**b**) TEM of MSI enterocytes 24 h after *E. coli* K1 colonization at P2; intracellular bacteria within vesicles (), enterocyte nucleus (N) and cell border (black dashed line) are indicated. (**c**) Confocal micrographs of MSI sections from neonatal rats 24 h after *E. coli* K1 colonization at P2; sections stained for Enterobacteriaceae 16S rRNA (red) and DNA (grey); right panel shows magnified images of regions defined by blue insets in left panels; luminal (), intracellular () and translocated () bacteria are indicated. (**d**) Bacterial load within mesenteric lymphatic node tissue (MLN) harvested from P2 and P9 rats 24 h after initiation of colonization with *E. coli* A192PP, showing enumeration of *E. coli* K1 (phage K1E-susceptible). *n* = 23 animals for non-colonised and *n* = 24 animals for colonized pups; line is median, *P < 0.05 (Student’s *t*).

*E. coli* K1 bacteria transit from the GI tract to the blood with low frequency after attaining a threshold level in the gut^6^. We examined temporal and spatial relationships between colonizing *E. coli* A192PP and cell populations in the small intestine and colon 24 and 48 h (Fig. 3a) after initiation of colonization. Sections were fixed to preserve the integrity of the mucus layer and colonizing bacteria were visualized with O18 polyclonal antibody. No bacteria reacted with the antibody in GI tissues from non-colonized P2 and P9 neonates, indicating that bacteria expressing the O18 antigen were not part of the microbiota of these pups. Large quantities of O18 antigen, representing *E. coli* A192PP, were found in the colon 24, 48 and 60 h after colonization of P2 and P9 pups. At these time points, O18-positive bacteria were maintained at a distance from the apical surface of the epithelium in all regions of the small intestine of animals colonized at P9. In contrast, large numbers of O18-positive bacteria were seen in close proximity to the gut epithelium in the MSI at 24, 48 (Fig. 3a) and 60 h (not shown) after colonization at P2. External surfaces of the villi in the MSI of animals administered *E. coli* A192PP at P2 were colonized with *E. coli* K1 (Figs 3b and 4a). At 24 and 48 h bacteria were located within intracellular vesicles of the villi (Fig. 3c) and progressed through the epithelial barrier by the transcellular route between 24 and 60 h after colonization (Fig. 4b,c). A high proportion of O18 LPS stain was associated with rod-shaped elements, but a significant fraction appeared with a diffuse staining pattern (Fig. 3c). FISH staining confirmed the presence of intact intracellular bacteria (Fig. 4c). Rod-shaped bacteria in contact with the epithelial surface were frequently observed by scanning electron microscopy of the mid small intestine of P2 but not P9 pups (Fig. 4a). Intact bacteria could also be visualized within intracellular compartments of enterocytes in this region of the GI tract using transmission electron microscopy (Fig. 4b). This data indicates that the MSI region provides a transcellular portal of entry into the blood circulation and this conclusion is supported by the presence of significantly greater numbers of *E. coli* K1 bacteria in mesenteric lymphatic tissue from P2 compared to P9 colonized rat pups (Fig. 4d).

### Paneth cell ablation and susceptibility to *E. coli* K1 infection

**Figure 5:**
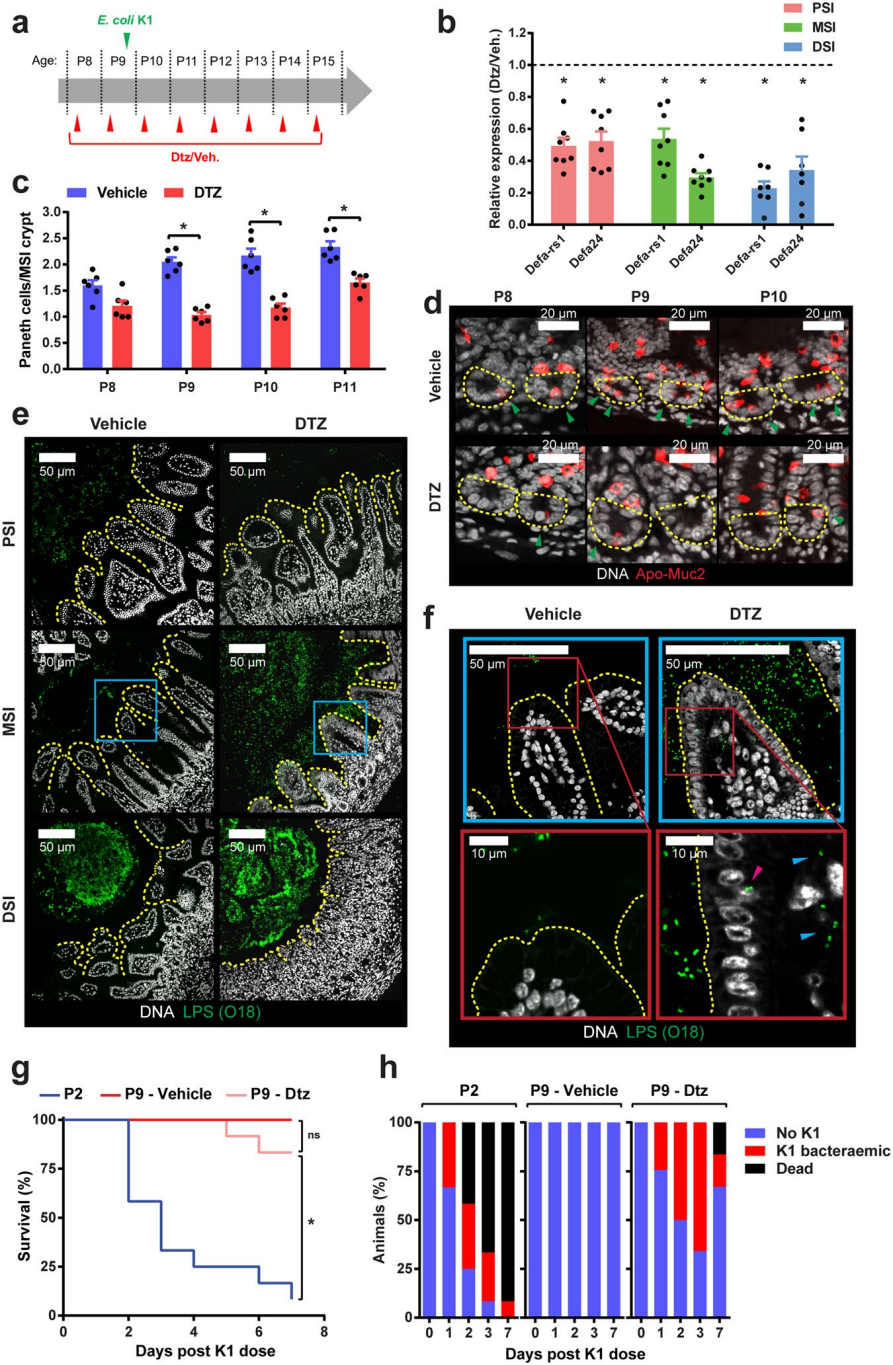
DTZ-mediated suppression of Paneth cell function in infection-resistant neonatal rat pups facilitates *E. coli* K1 invasion of the MSI. (**a**) Neonatal rats received 40–50 mg/kg DTZ or Li_2_CO_3_ buffer (vehicle) by i.p. injection as indicated. Colonization with *E. coli* A192PP initiated at P9. (**b**) Determination by qRT-PCR of *defa-rs1* and *defa24* in PSI, MSI and DSI 72 h after initiation of DTZ dosing, relative to values obtained with corresponding tissues from animals dosed with Li_2_CO_3_ buffer alone (dashed line); *n* = 7–8 animals, mean ± SEM (DTZ vs. buffer), *P < 0.05 (Mann-Whitney *U*). (**c**) Quantification of Paneth cells in crypts of MSI tissues over period P8-P11. Tissues were removed from rat pups that had been dosed with DTZ or Li_2_CO_3_ buffer according to schedule A; *n* = 6 animals, mean ± SEM, *P < 0.05 (Sidak’s multiple comparison). (**d**) Representative confocal micrographs used to calculate data in C; Methacarn-fixed MSI sections were stained for Apo-Muc2 (red) and DNA (grey); Paneth cells () and crypt base (yellow dashed line) indicated. (**e**) Confocal micrographs of Methacarn-fixed PSI, MSI and DSI sections obtained from pups dosed with DTZ or Li_2_CO_3_ buffer 48 h after initiation of colonization with *E. coli* A192PP at P9; sections stained for O18 LPS (green) and DNA (grey). (**f**) Magnification of stained MSI tissues corresponding to blue boxed regions indicated in E. Intracellular () and translocated () *E. coli* K1 indicated. Survival (**g**) and presence of *E. coli* K1 in blood (**h**) of neonatal rat pups colonized with *E. coli* A192PP. Pups (*n* = 12 animals for each group) were fed bacteria at P9 and dosed with DTZ or Li_2_CO_3_ buffer according to the dosing schedule (**a**). Response of P2 pups to colonization (no DTZ) is also shown.

Few Paneth cells are present in the small intestine at birth but numbers increase substantially with age^20^. Bacteria induce these cells to secrete substantial amounts of α-defensins^21^, which serve as characteristic markers of Paneth cell activity. Paneth cells are rich in zinc^22^ and bind diphenylthiocarbazone (DTZ), a cytotoxic compound with high affinity for zinc that selectively ablates Paneth cells in neonatal rats^23^. We used DTZ-mediated Paneth cell ablation in Sprague Dawley pups (Supplementary files) to determine the contribution of mucus-embedded antimicrobial α-defensins in protection against *E. coli* K1 systemic infection. Pups received 40–50 mg/kg DTZ at P8 and a second dose at P9 followed by oral challenge with *E. coli* A192PP 6 h later (Fig. 5a); maximal Paneth cell destruction occurs around 6 h after DTZ administration^24^. As Paneth cells begin to regenerate 24 h after DTZ administration^24^, pups were dosed with DTZ every 24 h until the scheduled end point. Paneth cell ablation in the small intestine was monitored by determination of *defa-rs1* and *defa24* expression with qRT-PCR. Levels of both α-defensins were suppressed by approximately 40% at 6 h after the initial DTZ dose. Suppression of *defa-rs1* and *defa24* rose to 60–70% at 20 h and these levels were maintained for at least three days after initiation of *E. coli* A192PP colonization of DTZ-treated P9 pups (Fig. 5b). Administration of DTZ significantly decreased the number of Paneth cells located in MSI crypts over the P8-P11 period (Fig. 5c), as determined by confocal microscopy following staining of small intestinal tissue sections with antiserum targeting the Paneth and goblet cell-specific Muc2 precursor form (Fig. 5d). Stained tissue sections revealed that DTZ treatment did not affect the number of goblet cells or the mucus layer (Fig. S2a–c). H&E stained sections showed that DTZ treatment did not cause any inflammation in the PSI or MSI, although transient epithelial shedding and inflammation was observed in DSI sections (Fig. S2d).

DTZ administration had no impact on the spatial relationship between colonizing *E. coli* K1 and the epithelial surface in the PSI or DSI (Fig. 5e). In contrast, DTZ exposure enabled colonizing bacteria to gain access to the enterocyte surface in the MSI (Fig. 5e,f). In similar fashion to *E. coli* A192PP-colonized P2 animals, bacteria were located between the villi of the MSI 48 h after initiation of colonization and were detected within intracellular vesicles of MSI enterocytes. *E. coli* K1 was observed in the *lamina propria*, indicating bacterial translocation across the epithelial barrier in this region of the GI tract (Fig. 5f). Survival of pups in DTZ and control groups was high (83.4% and 100% respectively) over the seven day observation period but in contrast to the control group 75% of animals receiving DTZ developed *E. coli* K1 bacteraemia (Fig. 5g,h).

### Maturation of the neonatal mucus barrier coincides with resistance to *E. coli* K1 infection

**Figure 6:**
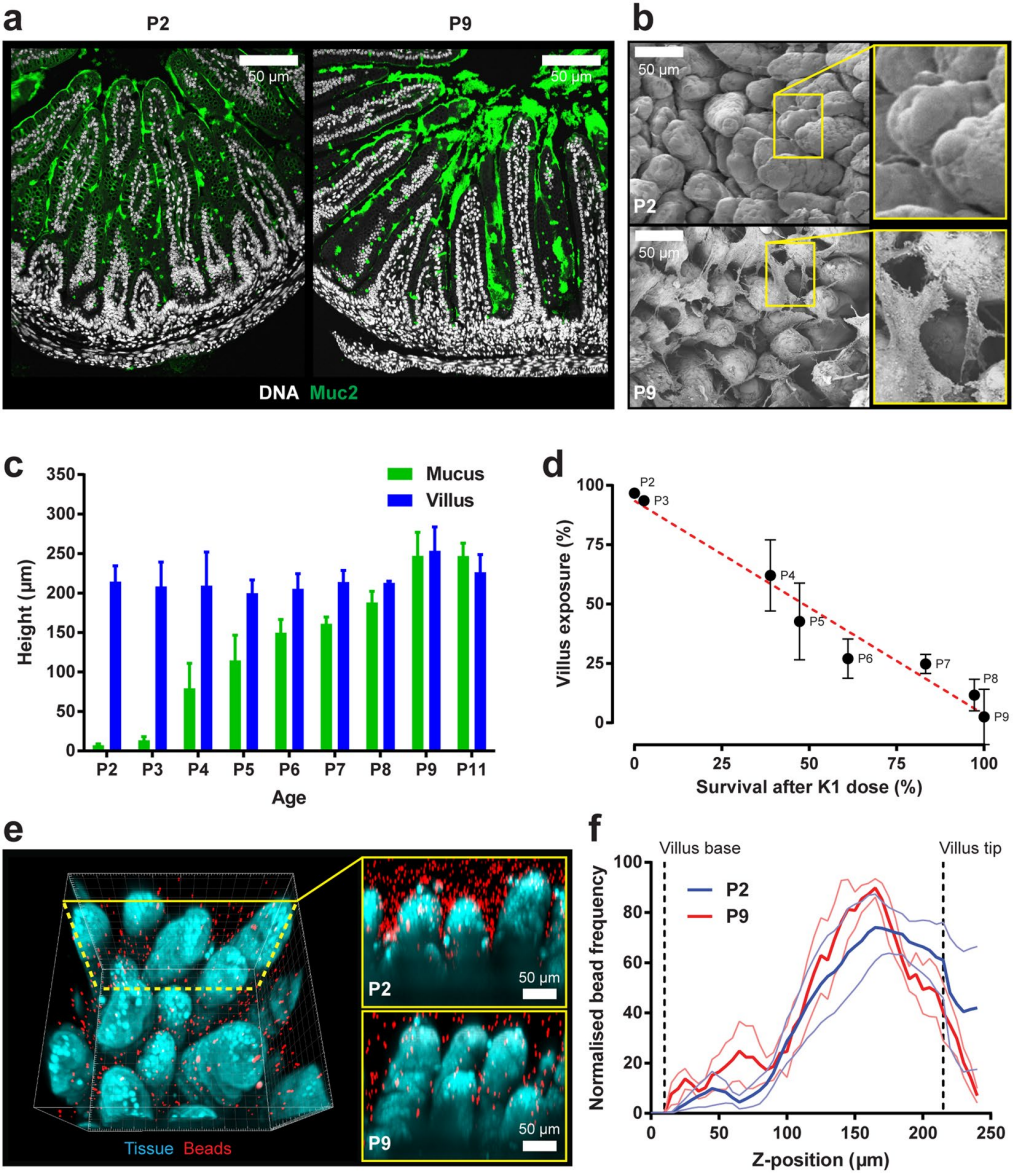
Postnatal MSI mucus layer maturation correlates with increased resistance to *E. coli* K1 systemic infection. (**a**) Confocal micrographs of Methacarn-fixed MSI tissue sections from neonates at P2 and P9; sections stained for Muc2 (green) and DNA (grey). (**b**) SEM of neonatal MSI tissue obtained at P2 and P9; right panels show magnification of inset yellow boxes in left panels. (**c**) *Ex-vivo* quantification of MSI mucus layer and villus height from P2-P11; *n* = 4 animals, mean ± SEM. (**d**) Correlation between MSI villus exposure (proportion of villus not covered by mucus layer) and survival between P2-P9 after initiation of *E. coli* A192PP colonization at P2; *n* = 4 animals, mean ± SEM, linear regression of data indicated by red dashed line. (**e**) Confocal z-plane projection of fluorescently labelled *ex-vivo* MSI tissue overlaid with fluorescent beads; representative 3D projection (left panel) and x/z-axis cross-sections from P2 and P9 tissues (right panels; yellow box) shown; labelled tissue (blue), fluorescent beads (red). (**f**) Quantification of z-axis bead distribution in mucus of MSI tissue from P2 and P9 neonates; data from confocal z-plane projections as shown in (**e**); error bars are SEM from *n* = 4 animals, mean ± SEM (thin lines); approximate positions of the villus base and tip along the z-axis are indicated (dashed lines).

All P2 pups succumb to invasive infection with *E. coli* A192PP following GI colonization (Figs 1a and 5g) but become progressively more resistant over the P2-P9 period^16^. Although we have shown that disruption of Paneth cell maturation allows *E. coli* K1 to invade the P9 MSI, secreted antibacterial peptides are primarily localized within the mucus of the small intestine^25, 26^. Little is known of early postnatal development of the mucus barrier or of goblet cells, the primary secretors of Muc2^27^. Fixation of MSI tissue in Methacarn for preservation of mucus followed by immunostaining revealed a substantial increase in secreted Muc2 between P2 and P9 (Fig. 6a), a finding supported by detection of mucus-like extracellular material in P9 but not P2 MSI tissue imaged by SEM (Fig. 6b). We therefore hypothesized that the protective small intestinal mucus layer was absent in *E. coli* K1-susceptible P2 neonates, but present in resistant P9 neonates. To address this, the thickness of the MSI mucus layer over the P2-P11 period was measured using an *ex vivo* explant system^28^. No mucus was detected in P2-P3 MSI. However, mucus began to fill the inter-villi spaces at P4 and increased progressively in thickness over P4-P9 until it reached the tips of the villi (Fig. 6c), demonstrating that small intestinal mucus layer formation occurred in the post-natal period and was incremental in nature. Importantly, the timeframe of mucus layer formation almost perfectly matched the period over which neonatal rats become resistant to *E. coli* K1 infection. Plotting survival of neonatal rats orally dosed with *E. coli* K1 at different ages from P2-P9 against villus exposure (the proportion of the villus not covered by the mucus layer) resulted in a highly significant negative linear correlation (Fig. 6d).

The small intestinal mucus is normally penetrable to bacteria or beads the size of bacteria, unlike colonic mucus which acts as an impenetrable physical barrier to bacteria. To test the penetrability of the neonatal small intestinal mucus, *ex vivo* MSI explants were overlaid with 1 µm fluorescent beads and imaged by confocal microscopy (Fig. 6e,f). Analysis of the spatial localization of beads between the villi showed a similar distribution pattern in explants from P2 and P9 animals, indicating that the neonatal small intestinal mucus remained penetrable to bacteria-sized beads.

**Figure 7:**
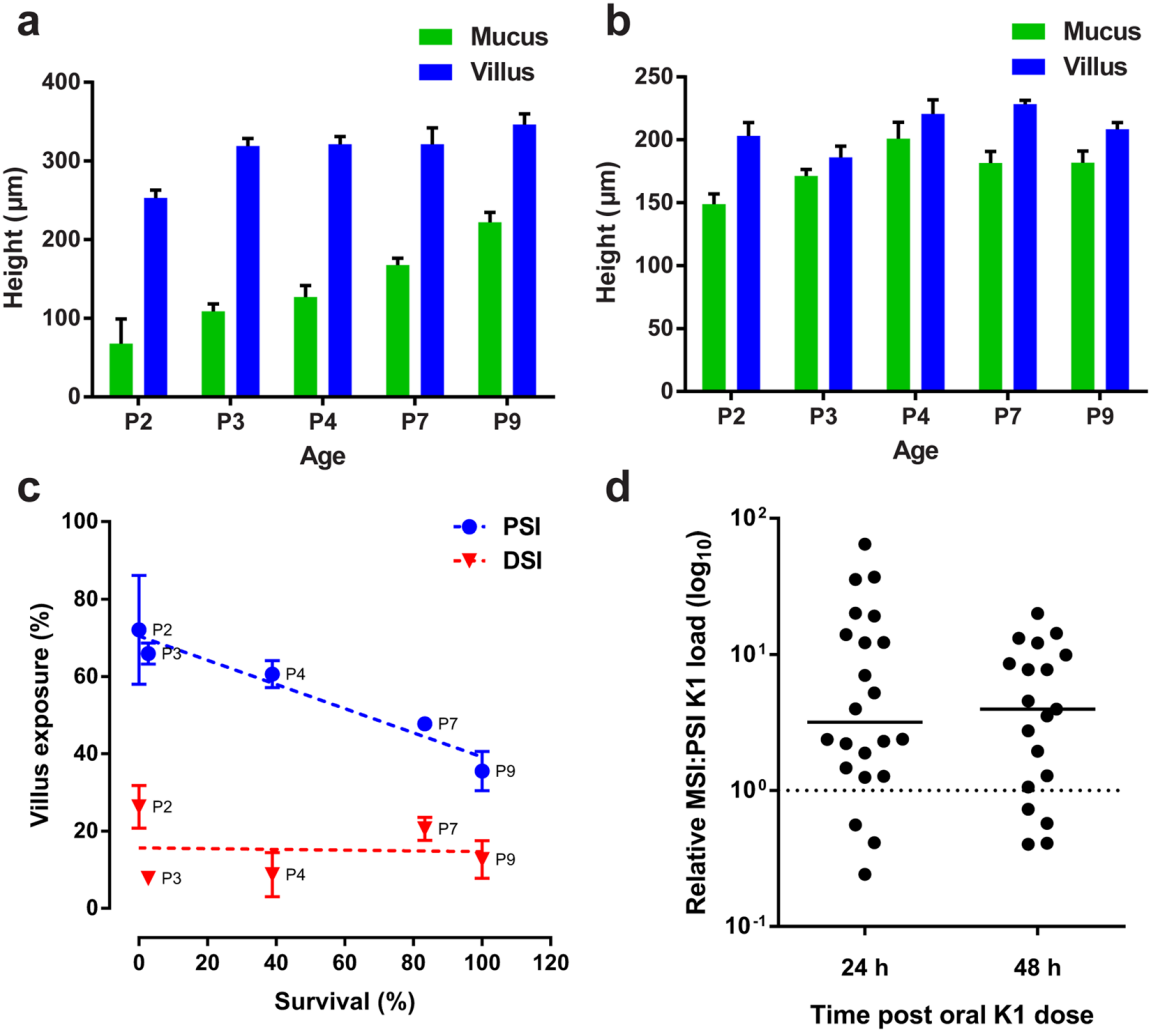
DSI mucus layer maturation and PSI bacterial load may form the basis of *E. coli* K1 invasive region-selectivity. *Ex-vivo* quantification of PSI (**a**) and DSI (**b**) mucus layer and villus height from P2-P11; *n* = 4 animals, mean ± SEM. (**c**) Correlation between PSI and DSI villus exposure (proportion of villus not covered by mucus layer) and survival between P2-P9 after initiation of *E. coli* A192PP colonization at P2; *n* = 4 animals, mean ± SEM, linear regression of data indicated by dashed lines. (**d**) Quantification of relative *E. coli* K1 load in the MSI compared to PSI (represented by dashed line) of neonatal animals 24 and 48 h after colonization at P2; line is median, *n* = 19–22 animals per group.

To shed light on the basis of the regio-selectivity of translocation across the GI epithelium, the development of the PSI and DSI mucus layers over the P2-P9 period was examined using the same *ex vivo* approach. Formation of the PSI mucus layer was gradual, with villus tips remaining exposed at P9 (Fig. 7a). In contrast, development of the DSI mucus layer was extremely rapid, with the villi covered in mucus by P3 (Fig. 7b). Plotting villus exposure in the PSI and DSI against susceptibility to *E. coli* K1 infection showed no correlation in the DSI, but a significant negative correlation in the PSI (Fig. 7c). This data suggested that a differential mucus layer maturation rate could explain the inability of *E. coli* K1 to invade the neonatal DSI tissue, but could not account for the lack of PSI invasion. Translocation of colonizing *E. coli* K1 from gut lumen to blood circulation is very inefficient, with systemic infection often due to a single bacterial cell that has traversed the gut epithelium and escaped capture by the mesenteric lymphatics^6^. There is likely to be a threshold level of *E. coli* K1 colonizers in the small intestine required to facilitate translocation and infection^6, 29^. Determination of the relative numbers of *E. coli* A192PP in the MSI and PSI of individual animals colonized at P2 revealed a median three- to fourfold difference at both 24 and 48 h in the ratio MSI:PSI after bacterial dosing (Fig. 7d), strongly suggesting that whilst the mucus layer in the PSI is not developed sufficiently to prevent *E. coli* K1 from gaining access to the epithelial surface, colonizing bacteria do not attain the threshold level necessary to effect epithelial translocation.

### *E. coli* K1 invasion occurs following removal of mucus

**Figure 8:**
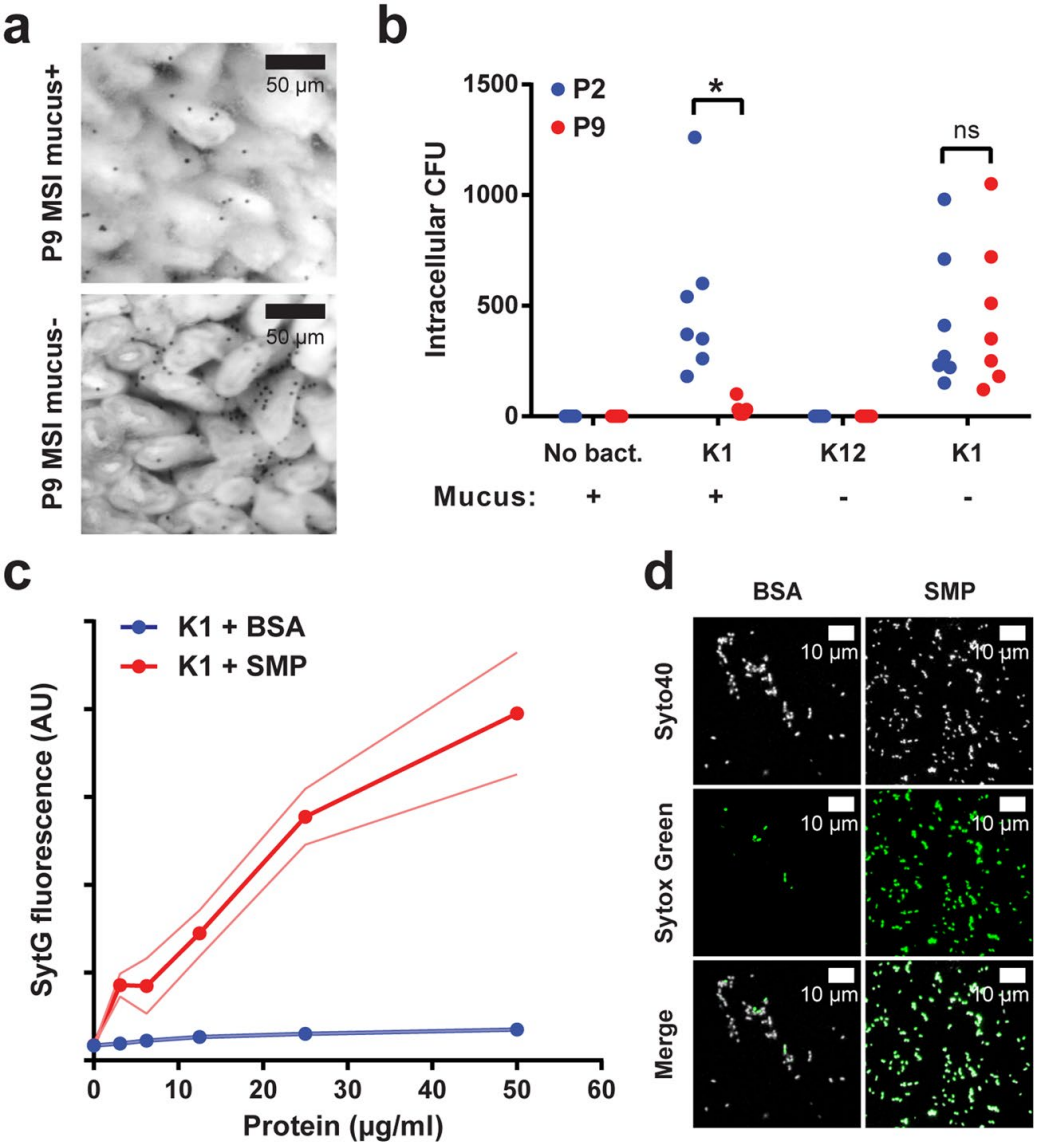
The mucus layer prevents *E. coli* K1 invasion of neonatal MSI tissue. (**a**) Bright-field micrograph of *ex-vivo* P9 neonatal MSI tissue overlaid with 10 µm black microspheres to visualize the mucus surface; tissue shown before (upper panel; mucus+) and after (lower panel; mucus−) mucus removal. (**b**) *Ex-vivo* tissue invasion: MSI tissues from P2 (blue) and P9 (red) neonates prepared with the mucus layer intact (+) or removed (−) as shown in panel a; tissues incubated with *E. coli* K1 or *E. coli* K12, intracellular bacteria isolated by gentamicin protection and quantified by plating; *n* = 7 animals for each group, *P < 0.05; n.s. = not significant (Sidak’s multiple comparison). (**c**) *E. coli* K1 membrane permeability: quantification of membrane-impermeable Sytox Green (SytG) fluorescence after exposure of *E. coli* K1 cells to bovine serum albumin (BSA; blue) or soluble mucus protein (SMP; red) extracted from P9 neonatal MSI mucus; error bars (thin lines) are SEM; *n* = 3 protein preparations. (**d**) Confocal micrographs of *E. coli* K1 exposed to 50 µg/ml BSA (left panels) or SMP (right panels) as detailed in panel c; cells were stained with membrane permeable Syto40 (upper panels; grey) and membrane impermeable Sytox Green (middle panels).

Mice deficient in Muc2 lack a mucus layer and bacteria are in contact with the intestinal epithelium in the colon^14^ and in the small intestine, where bacteria can be found between the villi and near the crypt openings (Birchenough, unpublished). As *Muc2* ^−/−^ rats are currently not available, the role of mucus in enabling resistance to *E. coli* K1 infection cannot be directly ascertained. However, by manual disruption of the mucus layer in isolated tissues we were able to determine the effect of mucus removal on the capacity of *E. coli* K1 to invade intestinal tissue *ex vivo*. Chamber-mounted P2 and P9 MSI tissues with intact or disrupted mucus (Fig. 8a) were exposed to *E. coli* A192PP or non-invasive *E. coli* K12 for 1 h. Intracellular bacteria were then quantified by gentamicin protection (Fig. 8b); no resident gentamicin resistant or intracellular bacteria were present in non-disrupted tissue samples that had not been exposed to these bacteria. No intracellular bacteria were found in tissues with disrupted mucus that were exposed to *E. coli* K12. In contrast, intracellular bacteria were cultured from tissue with intact mucus exposed to *E. coli* K1 and significantly more bacteria were found in P2 compared to P9 explants. Critically, disruption of the P9 mucus layer allowed *E. coli* K1 to invade P9 tissue to the same extent as P2 tissue.

Soluble mucus proteins (SMP) were extracted from the P9 explants and examined for bacteriolytic activity by incubation with *E. coli* K1 in the presence of the membrane-impermeable vital dye Sytox Green (Fig. 8c). A concentration-dependent increase in Sytox Green fluorescence was observed in cells treated with SMP but not BSA, confirming the presence of bacteriolytic factors in the SMP. When the bacteria were stained with the cellular dye Syto40, a high proportion of Sytox Green positive cells were observed after exposure to SMP compared to BSA (Fig. 8d). These results support the contention that the mucus layer and the antimicrobial factors it contains are the primary factors preventing access to and invasion of the MSI epithelium by *E. coli* K1. Thus, postnatal development of the small intestinal mucus layer is likely to play a key role in the determination of age-dependent resistance to systemic *E. coli* K1 infection.

## Discussion

A striking facet of human neonatal bacterial meningitis is the remarkably strong age dependency of these invasive infections. The two main etiologic agents, *Streptococcus agalactiae* and *E. coli* K1, are common components of the healthy adult microbiota but their vertical transmission to newborn infants carries the risk of severe systemic disease. That this age dependency can be readily replicated in the rat suggests that the capacity of these bacteria to cause neonatal disease is linked to anatomical, physiological or immunological features associated with the early life of the host.

In the rat, *E. coli* K1 colonization of the GI tract during the early neonatal period elicited changes in expression of genes involved in gut maturation. Downregulation of *Tff2* mRNA expression, accompanied by a decrease in colonic Muc2^16^, contrasts with reports of up-regulation of mucus gene expression induced in mice by colonization with commensals such as *Lactobacillus acidophilus* ^30^ and raises the question as to whether *E. coli* K1 causes more generalized disruption of the epithelial barrier to bacterial invasion. We established that *E. coli* K1 colonization at P2 and P9 does not lead to decreased epithelial barrier function, although general intestinal permeability was significantly higher in P2 compared to P9 pups. In this context, it has been determined in rats^18^ and in infants^31^ that intestinal permeability is higher in the first days of life. Our results indicate that it is unlikely that particulates, including bacteria, gain access to the submucosa as a result of *E. coli* K1-induced changes in gut permeability and a more selective mechanism is likely involved in *E. coli* K1 translocation across the epithelium.

The bulk of GI bacteria, including colonizing *E. coli* A192PP^16^, are found in the colon and are separated from the colonic epithelial surface by a two-layered mucus system, where the stratified inner layer physically separates the bacteria from the epithelial cells^14^. The outer colonic mucus layer is formed from the inner layer by the action of proteases and in its expanded nature is penetrable by resident bacteria. The colon is not the site of translocation from gut to blood circulation of colonizing *E. coli* K1 in the susceptible rat as the bacteria are maintained at a safe distance from the epithelial layer (Fig. 3a).

In contrast, the small intestine is protected by a single layer of mucus that, unlike the inner colonic mucus layer, contains pores that allow the penetration of bacteria and bacteria-sized particles^12^. We found very few bacteria in close proximity to the epithelial cell surface in the PSI and DSI. In contrast, 24 h after initiation of colonization of P2 pups large numbers of O18-positive bacteria were observed in close proximity to the epithelial surface of the MSI, within intracellular enterocyte compartments by 48 h (Fig. 3a) and subsequently within the mesenteric lymphatic system (Fig. 3d). As neither O18 LPS nor intracellular Enterobacteriaceae were observed in the non-colonized MSI, we are confident that the intracellular bacteria in this region of the GI tract of colonized animals were *E. coli* A192PP cells. Presumably a small number^6^ were able to circumvent the filter function of the lymph nodes to gain entry to the blood circulation. We did not investigate mechanisms of cellular uptake of *E. coli* A192PP but it is well established that macropinocytosis occurs in the GI tract of neonatal rats up to approximately 18 days *postpartum* ^32, 33^. This is supported by the numerous vacuolar enterocytes observed in the MSI and DSI and suggests that uptake of *E. coli* K1 proceeds through the macropinocytic pathway^34^. Indeed, some pathogens, including enterohemorrhagic *E. coli* ^35^ and *Yersinia pseudotuberculosis* ^36^, are known to utilize macropinocytosis to cross the intestinal epithelium following colonization of the GI tract. Failure of *E. coli* A192PP to translocate at the PSI or DSI could be partly due to absence of such pathways at these locations.

We previously showed that P9, but not P2, pups respond to the threat from *E. coli* A192PP colonization by upregulating genes encoding Paneth cell-derived α-defensins^16^. Now we show that partial ablation of Paneth cells in resistant P9 pups enables the pathogen to make contact with the epithelial surface in the MSI as observed in susceptible P2 pups, and to cause bacteraemia in a significant proportion of animals (Fig. 4). Only a limited number of deaths occurred in DTZ-treated pups, indicating that although bacteraemia is lethal in naturally susceptible P2 pups, other factors determine survival in the older neonates. Partial Paneth cell ablation has also been shown to sensitize P4 neonatal rat pups to *E. coli* O18:K1:H7 strain Ec5 with significant increases in the number of *E. coli* in the GI tract, mean illness scores and deaths^23^. Examination of ileal sections from preterm infants with necrotizing enterocolitis revealed low numbers of Paneth cells compared to age-matched samples from infants with spontaneous intestinal perforations; replicating this Paneth cell loss with DTZ treatment of P14-P16 mice combined with exposure to *Klebsiella pneumoniae* induced a similar pathological state^37, 38^. Such studies emphasize the parallels between experimental animal models and human disease in GI tract homeostasis.

Our investigation of mucus maturation in the MSI over the critical period P2-P11 (Fig. 6) provides a physiological basis for the susceptibility of neonates to infection and subsequent development of resistance to disease. MSI mucus displays significant antibacterial activity and it is likely that a proportion of the free LPS that we detected in GI sections represent debris from bacteria lysed by antibacterial peptides confined within the mucosal barrier. Villi in this region of the GI tract almost certainly make frequent contact with luminal bacteria, including colonizing *E. coli* K1, due to the inadequate protection provided by the thin, developing mucus layer. The newborn face a number of complex and sometimes conflicting immunological demands, including avoidance of excessive inflammatory responses to bacteria and their products, that may leave them at risk of infection^39^. An immature immune system means the neonatal host must rely to a large extent on innate immunity to repel would-be pathogens; our study demonstrates the importance of a mature antibacterial mucus barrier.

As with the MSI, villi in the PSI remain exposed as the mucus layer matures in this region of the small intestine but the burden of colonizing *E. coli* K1 cells in the PSI is significantly lower than in the MSI. As colonizers of the small intestine need to achieve a threshold level in order to make sufficient contact with epithelial surfaces prior to translocation, we hypothesise that the number of colonizing bacteria in the PSI is insufficient to effect epithelial traversal and translocation to the blood compartment. On the other hand, the mucus layer in the DSI matures early to protect the epithelium and ensures that no bacterial contact with the epithelial surface occurs. These observations would appear to provide a rationale for the regio-selective susceptibility to *E. coli* K1 invasion from the neonatal GI tract.

Penetration of small intestinal mucus by bacteria and other microparticles is poorly understood. Nutrient uptake is localized to this region of the intestine and a relatively porous mucosal layer may be functionally relevant. There is evidence that lack of close contact between bacteria and epithelial cells in adult animals is due to antibacterial peptides and proteins produced by Paneth cells (for example, ref. 12). The present study shows for the first time that partial Paneth cell ablation, leading to a lower concentration of antibacterial peptides, enables bacteria to gain access to the epithelium, even in the presence of an intact mucus layer. On the other hand, in the absence of mucus the antibacterial peptides will quickly diffuse into the lumen and have limited effects where most needed, close to the epithelial surface. The mucus provides the necessary diffusion barrier for the antibacterial peptides and also slows bacterial penetration. Thus, the antibacterial components and mucus act cooperatively to protect the small intestine. The poorly developed neonatal MSI mucus layer likely enables transcytosis and invasion by the *E. coli* K1 neuropathogen. Our proposed model for *E. coli* K1 and MSI interaction in the neonatal rat is shown in Fig. S3.

## Methods

### Colonization of neonatal rats

Animal experiments were approved by the Ethical Committee of University College London and were conducted in accordance with national legislation under United Kingdom Home Office Project License PPL 70/7773. All experiments were conducted in accordance with the United Kingdom Animals (Scientific Procedures) Act, 1986 and the Codes of Practice for the Housing and Care of Animals used in Scientific Procedures, 1989. All animal experiments were undertaken with *E. coli* K1 A192PP^7^. Either pathogen-free Wistar (Harlan) or Sprague-Dawley rat litters (Charles River) were used. Litters comprised 10–14 pups and were retained in a single cage with their natural mothers. The animal model of colonization and infection has been described in detail^17^. Each pup was fed 20 μl of Mueller-Hinton (MH) broth culture of *E. coli* A192PP (2–6 × 10^6^ CFU, 37 °C) from an Eppendorf micropipette. Controls were fed 20 μl of sterile broth. Colonization was determined by MacConkey agar culture of perianal swabs and bacteraemia by culture of daily blood samples taken from the footpad. *E. coli* K1-specific lytic bacteriophage K1E was administered orally to *E. coli* K1-colonized rat pups; an oral bolus of 10^9^ PFU of phage K1E reduced the number of *E. coli* K1 bacteria in the GI tract to undetectable levels.

### Bacterial enumeration by qPCR

Quantitative PCR was employed to quantify the bacterial constituents of the GI microbiota and to enumerate *E. coli* K1 in GI tissue segments. Total bacteria were estimated by amplification of 16S rDNA and quantification of *E. coli* K1 was based on amplification of K1-specific *neuS* ^16^.

### Intestinal permeability

The GI tract permeability of rat pups was assessed using FITC, FITC-dextran (Sigma) and 1 μm fluorescent microspheres (FluoSpheres; Molecular Probes). For FITC and FITC-dextran, pups were fed 15 μl of probe solution (800 mg/ml in sterile PBS) at 24 and 48 h after colonization or sham-colonization (with MH broth vehicle alone) and culled 1, 2 or 4 h after administration. For Fluospheres, pups were fed a 20 μl suspension of microspheres (containing 7.27 × 10^8^ beads) and animals culled up to 48 h later. Plasma fluorescence was determined against plasma from control rat pups, and values normalized for total blood volume for each pup.

### Tissue collection and processing

Neonatal rats were killed by decapitation. GI tissues were segmented into stomach, mesentery and colon, and 2 cm segments from proximal, middle and distal small intestine excised without washing^17^. Tissues were placed in methanol-Carnoy’s fixative (Methacarn) and maintained at room temperature for at least 3 h. For enumeration of *E. coli* K1 by viable counting and for extraction of DNA, tissues were placed in 2 ml ice-cold phosphate-buffered saline, homogenized and DNA extracted with QIAamp Stool DNA Mini Kit (Qiagen). For RNA extraction, tissues were transferred to RNAlater (Ambion), stored overnight at 4 °C and RNA extracted with RNeasy Midi Kit (Qiagen).

### Histology, immunohistochemistry and microscopy

Paraffin-embedded sections were dewaxed and hydrated; some sections were stained with H&E. Muc2 was stained using polyclonal rabbit antiserum raised against either non-glycosylated apo-Muc2 protein (PH497) or mature glycosylated Muc2 protein (Muc2C3); Alexa Fluor 555/488-conjugated goat anti-rabbit IgG (ThermoFisher) was used as secondary antibody. FISH was performed using Alexa Fluor 555-conjugated enterobacterial 16S probe 5′-CCC CCW CTT TGG TCT TGC-3′^14^ Immunofluorescent and FISH sections were counterstained using Hoechst 33258 (Sigma). *E. coli* A192PP was detected in tissue sections using rabbit polyclonal antibody against O18 LPS surface antigen^8^. Stained sections were mounted in Prolong Anti-fade (Life Technologies). Images were acquired using an LSM 700 confocal microscope equipped with an oil immersion lens and 488 nm and 555 nm lasers (Zeiss). Micrographs of GI tissues were obtained using a JEOL ISM-5300 scanning electron microscope; tissues were processed as described^40^.

### Selective ablation of Paneth cells

DTZ (10 mg/ml) was prepared in 25 mM Li_2_CO_3_^23^. Neonatal rats received 40–50 mg/kg DTZ by i.p. injection. The minimum bactericidal concentration of DTZ for *E. coli* K1 exceeded 4 mg/ml. Paneth cell ablation was determined by measurement of reduction of *defa-rs1* and *defa24* expression with quantitative RT-PCR using RNA extracted from GI tissue segments^16^. Tissues were collected from the small intestine, fixed, sectioned and examined as described above. Administration of DTZ did not alter the appearance of sectioned GI tract tissues (Fig. S2D).

### *Ex-vivo* characterization of the small intestinal mucus layer

Mucus was characterized using a tissue explant system^28^. Small intestinal tissues were dissected, flushed with Krebs buffer and mounted in a 1.5 mm diameter horizontal perfusion chamber. The mucus thickness was quantified by visualizing the mucus surface using apical addition of 10 µm black polystyrene beads and the distance between the mucus surface and villus base measured using a micropipette viewed through a stereomicroscope. Villus height was quantified by measuring the distance between the villus tip and base. Mucus penetrability was characterized as described^41^. Chamber-mounted tissue was stained with 15 µM Syto9 dye (ThermoFisher) for 15 min then overlaid with 1 µm crimson Fluosphere beads (ThermoFisher). Beads were allowed to sediment for 10 min and tissue and beads visualized by generating confocal z-stacks using an LSM 700 confocal microscope equipped with a water immersion objective lens and 488 nm and 640 nm lasers (Zeiss). Fluorescence intensity data for individual z-planes was extracted from z-stacks using Zen software (Zeiss) and was used to determine bead distribution in relation to villus tip and base.

### *Ex-vivo* bacterial tissue invasion assay

Small intestinal tissue was mounted in a chamber and the mucus surface visualized using polystyrene beads as described above. In some tissues the mucus layer was disrupted and removed using a micropipette. Mucus removal was confirmed by reapplication of polystyrene beads. *E. coli* A192PP and K12 strain W3110 (10^6^ CFU) were applied apically to tissue explants and incubated at 37 °C for 1 h. Explants were washed with Krebs buffer, transferred to Krebs buffer with 100 µg/ml gentamicin (Sigma) and incubated for 1 h at room temperature. Tissues were washed twice (4 °C; 10 min), homogenized, and viable bacteria determined.

### Bacteriolytic activity of soluble mucus protein extracts

Mucus was collected into 10 mM sodium phosphate (SP) buffer from chamber mounted small intestinal explants using a micropipette. Insoluble material was removed, supernatants containing SMP recovered, and protein quantified using BCA assay kit (Pierce). The capacity of SMP to permeabilize *E. coli* K1 cells was assessed by Sytox Green uptake. Mid-log phase *E. coli* A192PP cells were suspended in 10 mM SP buffer with 1 µM Sytox Green (ThermoFisher) and incubated in the dark for 10 min. Bacteria were diluted 1:20 into 10 mM SP buffer containing 1 mM EDTA and 2 µM Sytox Green and 100 µl bacteria distributed into wells of a 96-well black plate; 100 µl dilutions of SMP were added to each well and incubated for 5 h at 37 °C with shaking. Sytox Green fluorescence was normalized to wells containing bacteria without SMP or BSA. To visualize cells, suspensions were incubated with 10 µM Syto40 dye for 15 min and 20 µl transferred onto a microscope slide, air dried, mounted and examined using a LSM 700 confocal microscope with an oil immersion objective lens and 405 nm and 488 nm lasers (Zeiss).

## Electronic supplementary material

Supplementary information
