# Supplementary material for: Postnatal development of the small intestinal mucosa drives age-dependent, regio-selective susceptibility to Escherichia coli K1 infection
Source: Sci Rep. 2017 Mar 6;7:83. doi: 10.1038/s41598-017-00123-w (PMC5427930; doi:10.1038/s41598-017-00123-w)
Supplement: Supplementary file 1 — Supplementary information [file 41598_2017_123_MOESM1_ESM.pdf]

Postnatal development of the small intestinal mucosa drives age-dependent, regio-selective susceptibility to *Escherichia coli* K1 infection

George M.H. Birchenough, Fatma Dalgakiran, Luci A. Witcomb, Malin E.V. Johannson, Alex J. McCarthy, Gunnar C. Hansson, and Peter W. Taylor

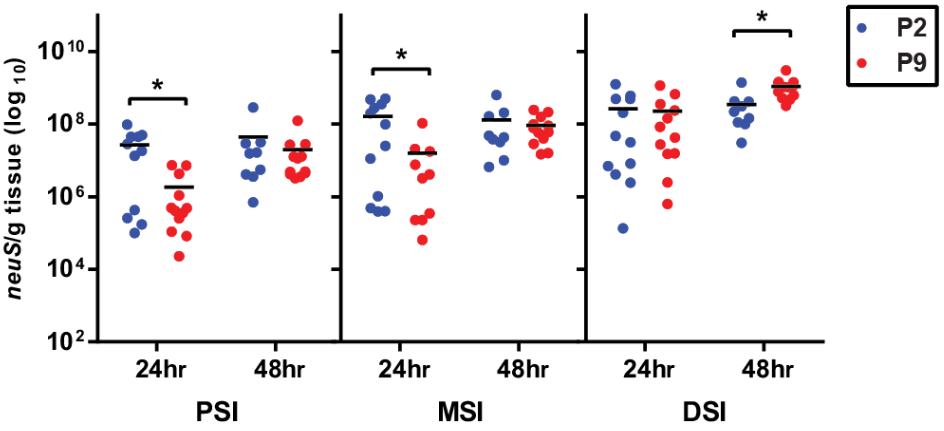

Figure S1|Colonization of neonatal rats following an oral dose of *E. coli* K1. Colonization of intestinal regions 24 and 48h after oral dosing of neonatal rats with *E. coli* A192PP at P2 or P9 was determined by qPCR quantification of *E. coli* K1-specific *neuS*; *n* = 4-12 animals; mean ± 1SEM, * $P < 0.05$. PSI, proximal small intestine; MSI, mid-small intestine, DSI, distal small intestine.

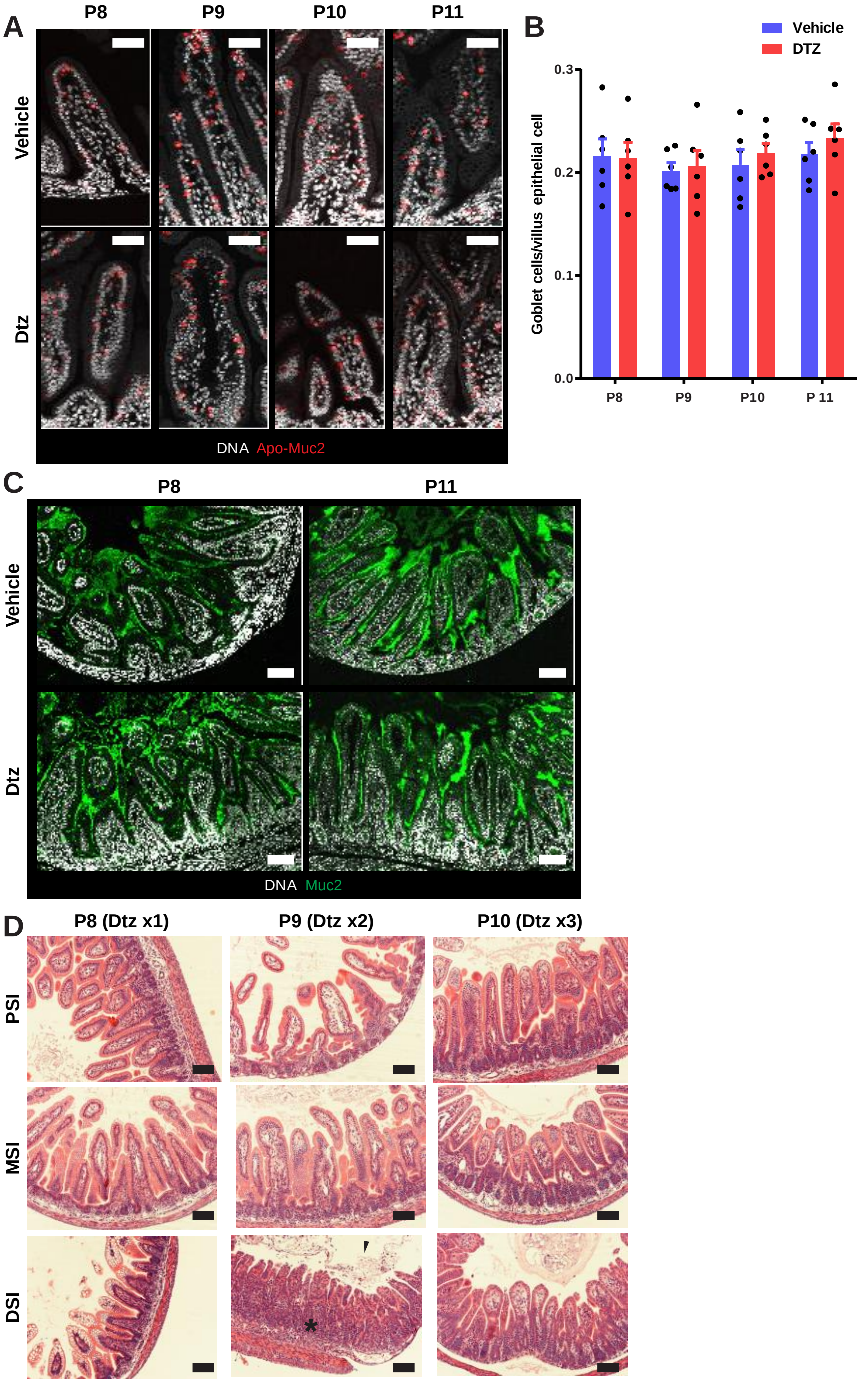

Figure S2 | Effects of dithizone (DTZ) treatment on the small intestine. Neonatal rats received 40-50 mg/kg DTZ or Li_2CO_3 buffer (vehicle) by i.p. injection from P8-P15 as indicated in Figure 5A. Small intestinal tissue was collected on different days and fixed in Methacarn (A) Representative confocal micrographs of MSI tissue stained to detect villus goblet cells; MSI tissue was stained for apo-Muc2 (red) and DNA (grey). (B) Quantification of MSI villus goblet cells based on images shown in (A); goblet cell number normalized to total epithelial cell number; n=6 pups per group, mean ± SEM. (C) Representative confocal micrographs of MSI tissue stained to detect the mucus layer; MSI tissue was stained for Muc2 (green) and DNA (grey). (D) Micrographs of H&E stained PSI, MSI and DSI tissue sections after 1 (P8), 2 (P9) and 3 (P10) DTZ injections; inflammation (*) and shed cells (→) in P9 DSI tissue is indicated. Scale bars 50 μm.

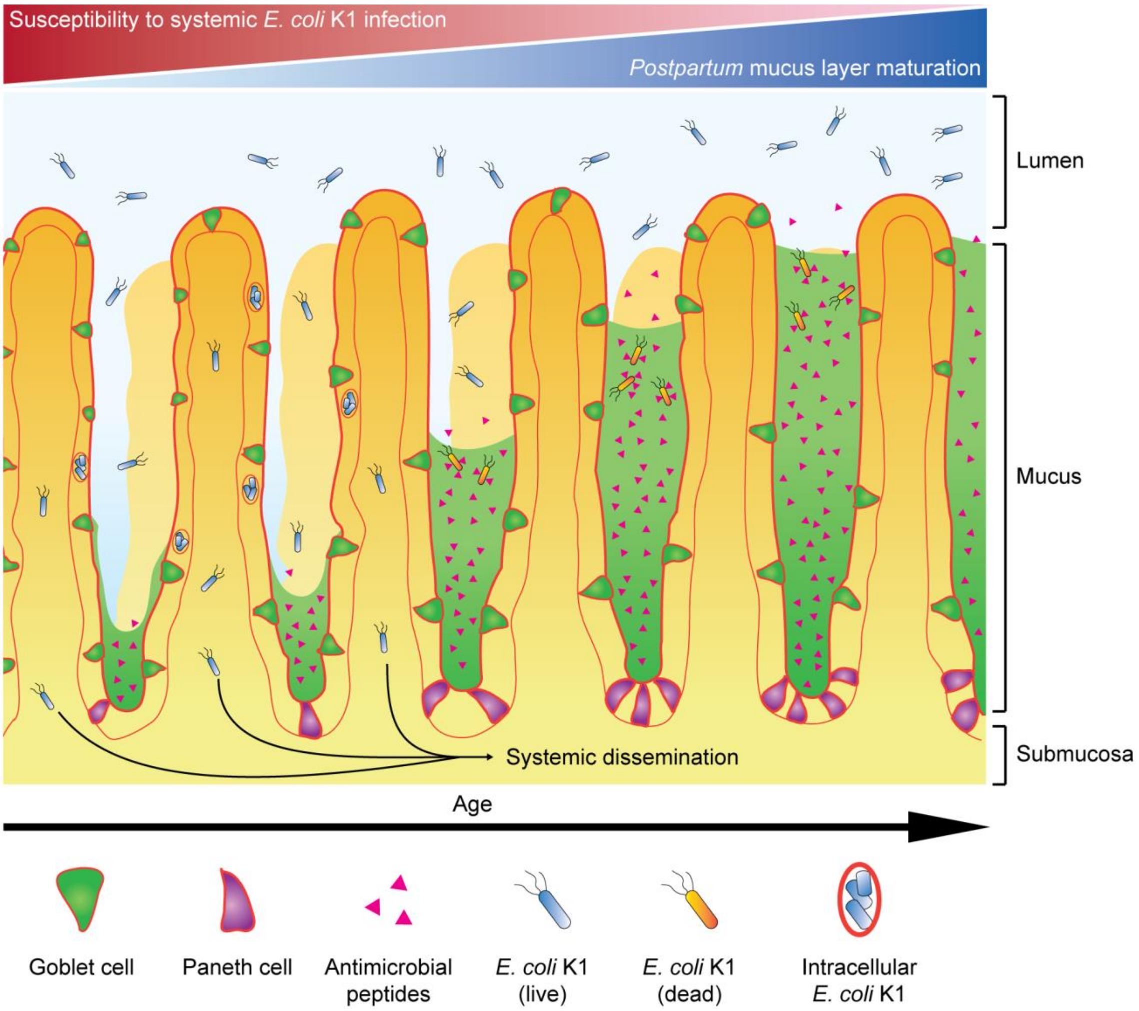

Figure S3 | Proposed model for transition from susceptibility to resistance to *E. coli* K1 infection in neonates following colonization of the GI tract.

Paneth cell ablation was performed in Sprague-Dawley, not Wistar rat pups

In initial experiments, Wistar rat pups, housed with the natural mother, received daily doses of 40-50 mg/kg DTZ, beginning at P8, by intraperitoneal (i.p.) injection. All animals receiving DTZ remained healthy but all mothers (4/4) died within 24 h of initiation of DTZ dosing of their offspring. Reducing the number of pups within a litter receiving DTZ did not prevent death of the mother rat. Lactating mothers lick the genital region of their offspring to stimulate digestion and we surmise that this led to ingestion of toxic metabolites of DTZ. We circumvented this effect by employing Sprague Dawley rat litters; with this rat strain, approximately half (10/18) of mothers survived when DTZ was administered to their offspring. Thus, all further DTZ experiments were conducted with Sprague Dawley rats. If the birth mother appeared unwell within 24 h of DTZ administration to the litter, the adult was culled and a surrogate Wistar mother, discharged from other experiments, was assigned to care for the pups; all surrogate mothers survived and successfully maintained the relevant litters. Patterns of GI colonization and survival profiles of Sprague Dawley pups were comparable to those obtained with Wistar rats.
